# An agentic framework for gravitational-wave counterpart association in the multi-messenger era

**DOI:** 10.1016/j.xinn.2026.101538

**Published:** 2026-08-11

**Authors:** Yiming Dong, Yacheng Kang, Junjie Zhao, Xinyuan Zhu, Ziming Wang, Lijing Shao

**Affiliations:** 1 Department of Astronomy, School of Physics, Peking University, Beijing 100871, China; 2 Kavli Institute for Astronomy and Astrophysics, Peking University, Beijing 100871, China; 3 Institute for Gravitational Wave Astronomy, Henan Academy of Sciences, Zhengzhou 450046, China; 4 School of Information Science and Technology, University of Science and Technology of China, Hefei 230026, China; 5 National Astronomical Observatories, Chinese Academy of Sciences, Beijing 100101, China

Dear Editor,

As a landmark discovery in astronomy and fundamental physics, GW170817 was the first binary neutron star (BNS) merger detected through gravitational waves (GWs) and accompanied by multi-wavelength electromagnetic (EM) counterparts.^1^ Joint observations of the GW signal, the associated short-duration gamma-ray burst (SGRB), and the kilonova transient have yielded remarkable scientific returns,^1^ including smoking-gun evidence for the BNS merger origin of SGRBs and kilonovae,^2^ and an independent measurement of the Hubble constant via the standard siren method.^3^ Besides BNSs, neutron star-black hole (NS-BH) mergers, as well as stellar-mass binary BH (BBH) mergers in active galactic nucleus (AGN) disks, have also been speculated to give rise to EM counterparts.^4^ Unfortunately, GW follow-up campaigns have so far been unsuccessful in identifying convincing EM counterparts to NS-BH or BBH mergers. The non-detections may result from the large distances of these events, incomplete coverage of their large GW localization regions, or intrinsically fainter EM emissions from them.

With the advent of next-generation GW detectors and advancing EM facilities, multi-messenger astronomy is expected to boost with richer scientific outcomes for all types of compact binary mergers discussed above. These opportunities, however, are accompanied by greater data-analysis challenges. In particular, searching for an association between GW signals and their EM counterparts is vital for enabling subsequent multi-messenger studies. The expected increase in co-detecting them will place growing pressure on both computational resources and human effort, preventing us from fully exploiting the scientific value of data.

The rapid advancement of artificial intelligence provides a promising pathway to help address emerging challenges. Machine learning has enabled real-time parameter estimation for BNS merger signals, delivering accurate estimates of sky localization within seconds,^5^ thereby supporting early alerts for multi-messenger follow-up. It has also been applied to searches for EM counterparts, assisting candidate prioritization in complex observational environments.^6^ Beyond conventional machine-learning approaches, the recent rise of large language models (LLMs) further shows the broad potential of artificial intelligence for scientific discoveries. Owing to their rapidly advancing reasoning capabilities, LLMs have been successfully explored in diverse scientific tasks, including automated scientific discovery.^7^ These LLM systems are able to achieve robust performance while substantially reducing the human effort and alleviating the pressure imposed by the rapidly growing data volume.

Motivated by this, we develop GW-Eyes, a first LLM-powered agentic framework designed to automate GW counterpart association tasks while assisting human experts through natural-language interaction with auxiliary functions such as catalog management, skymap visualization, and rapid verification. GW-Eyes integrates domain-specific tools tailored to the distinctive data structures of multi-messenger observations and dynamically retrieves and updates the latest information from the internet. We construct simulated datasets to evaluate the accuracy and efficiency of its tool use and further demonstrate its application to EM counterpart searches using real observational catalogs. Through GW-Eyes, we demonstrate how LLMs can participate in future multi-messenger observations and help address challenges in the big-data era.

## Framework overview

GW-Eyes is a general LLM-driven agentic system designed for GW counterpart association. As illustrated in Figure 1A, it comprises two sub-agents, Collector and Executor, and integrates domain-specific tools through the Model Context Protocol. Collector is responsible for data acquisition and initial preprocessing, including the retrieval of GW posterior information, public EM event catalogs, and real-time circulars from the General Coordinates Network (GCN). Executor operates on the database to perform counterpart association tasks as well as auxiliary functions such as visualization and rapid verification.

**Figure 1:**
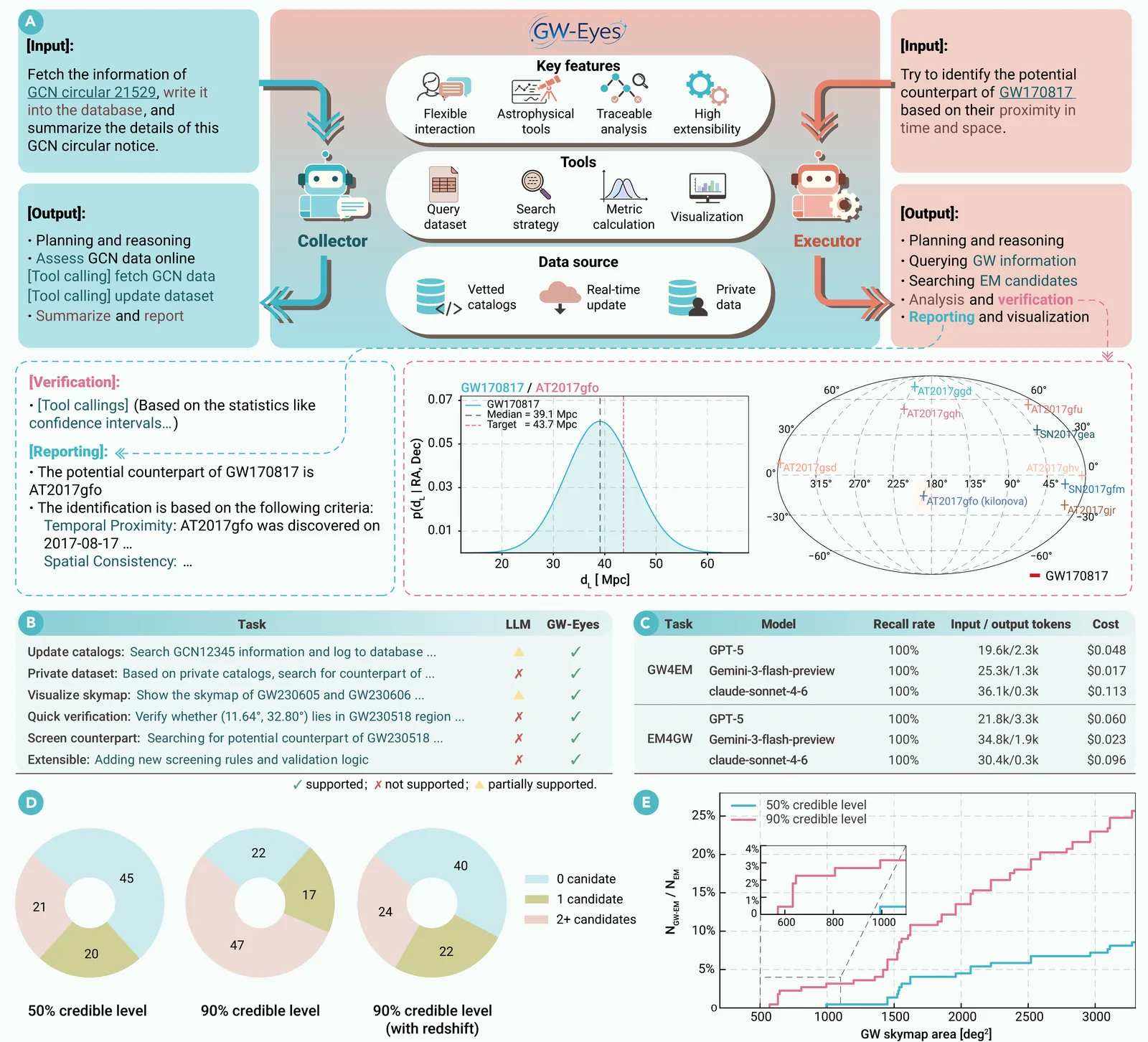
GW-Eyes enables autonomous GW counterpart association in multi-messenger astronomy (A) Based on a Collector-Executor sub-agent architecture with domain-specific tool integration, GW-Eyes performs counterpart association and related tasks through natural-language interaction. (B) Compared with general-purpose LLMs, GW-Eyes achieves improved adaptation to the specialized domain of multi-messenger astronomy. (C) GW-Eyes demonstrates stable performance and lower cost in evaluations on simulated datasets. (D) Using real catalogs, AGNFRC and GWTC-4.0, GW-Eyes performs preliminary associations based on temporal and spatial correlations. (E) The cumulative ratio *N*_GW-EM_/*N*_EM_ as a function of the GW skymap area threshold provides statistical insight into counterpart association and shows the growing data pressure expected from the increasing rate of future closely detected events.

Upon receiving a data-acquisition task, such as updating GCN circular records, Collector first retrieves the relevant online GCN circular contents (see Figure 1A). These records are typically human-generated text in which key event information, such as sky position and redshift, is embedded in natural language. Collector then automatically interprets and extracts the information relevant to counterpart association, performs rule-based and optional self-consistency checks, and stores it in the database according to predefined rules, thereby enabling subsequent analyses by the Executor.

For counterpart association tasks, such as searching for EM counterpart candidates to a given GW event, Executor first decomposes the request and formulates an analysis strategy. It then invokes tools to access GW posteriors and EM catalogs and evaluates their spatial associations using statistical metrics. After generating a list of candidates, Executor can provide visualizations, including sky localization maps and the luminosity distance posterior, together with a detailed report summarizing the selection criteria and final results. The association analysis can be performed in either direction: starting from a GW event to identify potential EM counterparts or from an EM transient to identify candidate GW events. With its modular agentic architecture, GW-Eyes provides a flexible framework that is not limited to a specific class of EM transients.

GW-Eyes can also assist human experts through natural-language interaction with auxiliary tasks, enabling faster and more convenient information retrieval and verification. Through natural-language queries alone, GW-Eyes can report the basic properties of EM events, visualize the posterior distribution of GW parameters, and rapidly determine whether a given coordinate lies within a high-confidence region of a GW event. In addition, as shown in Figure 1B, the agentic design of GW-Eyes and its integration of domain-specific tools provide clear advantages over general-purpose commercial LLMs. For instance, GW-Eyes can build and maintain a local event database with consecutive updates while supporting secure access to private data. In highly specialized multi-messenger astronomy, general-purpose LLMs often struggle to process domain-specific data effectively, whereas our integration of dedicated tools substantially improves the adaptability of GW-Eyes.

## Evaluation and application

To evaluate the performance of GW-Eyes on counterpart association, as well as the accuracy and efficiency of its tool use, we construct a simulated EM catalog for Gravitational-Wave Transient Catalog 4.0 (GWTC-4.0)^8^ and test whether GW-Eyes can correctly invoke relevant tools to recover the injected EM counterparts. Specifically, we select the first 50 GW events from GWTC-4.0. For each GW event, we inject one ground-truth counterpart satisfying the temporal, sky-position, and redshift criteria, together with two redshift-inconsistent confounders and ten temporally coincident unrelated events. All simulated EM events are then combined into a single catalog for retrieval tasks. We evaluate GW-Eyes on two tasks: “GW4EM,” which retrieves the injected EM counterpart for a given GW event, and “EM4GW,” which retrieves the associated GW event for a given EM event. During evaluation, GW-Eyes can freely invoke tools to complete each task and is required to provide its final answer in a predefined format. Figure 1C shows that GW-Eyes achieves stable performance on association tasks across different LLM back ends at relatively low cost.

We also demonstrate the application of GW-Eyes to EM counterpart searches using real catalogs. Given that most events in GWTC-4.0 are BBH mergers, we take AGN optical flares as an example to explore potential associations. We use the Active Galactic Nuclei Flare Refined Catalog (AGNFRC),^9^ whose temporal coverage overlaps with GWTC-4.0. We adopt a temporal window of 180 days after each GW trigger, apply GW-Eyes to search for potential temporal and spatial associations between events in the two catalogs, and report candidate counterparts whose sky position falls within the 50% and 90% credible regions of the GW localization. For events with reported redshifts, we further require consistency within the 68% credible region. The associations reported by GW-Eyes are independently cross-checked through manual execution of the tools, with consistent outcomes. Results are shown in Figure 1D. Given the relatively long time window adopted and poor sky localization of some GW events, temporal and spatial associations alone can yield more than one EM candidate for certain GWs. In addition, we compute the number of associated GW-EM pairs (*N*_GW-EM_) with different GW localization area thresholds, normalized by the number of EM events (*N*_EM_) within the adopted 180-day post-trigger temporal windows of the 50 GWTC-4.0 events. Here, the GW localization area for each event is defined as the sky area enclosed by the 90% credible interval. Figure 1E shows the cumulative ratio *N*_GW-EM_/*N*_EM_ as a function of the GW skymap area threshold. In the future, the Einstein Telescope is expected to detect $O\left(10^{4}\right)$-$O\left(10^{5}\right)$ BNS mergers per year and could enable the Rubin Observatory to detect 10 to 100 kilonovae annually.^10^ With the numbers of GW and EM events growing rapidly, counterpart association will become increasingly challenging. Fortunately, GW-Eyes can substantially help address these challenges and support different groups in tailoring its use to specific needs, greatly facilitating subsequent identification and follow-up studies.

## Interpretability and extensibility

Compared to other machine-learning-based approaches, GW-Eyes, built on an LLM agent framework, provides more interpretable reasoning traces and results. On the one hand, the full decision-making process, including tool usage and parameters, is recorded in human-readable natural language, facilitating inspection and verification. On the other hand, compared with general-purpose LLMs, the integration of domain-specific tools significantly reduces hallucinations in the specialized context of multi-messenger astronomy, therefore improving the accuracy and reliability of its results.

GW-Eyes is highly extensible. In the present study, counterpart association relies primarily on basic temporal and spatial overlap between GW and EM signals, while more specialized criteria can be readily incorporated by extending the agent’s toolset. For different EM datasets, this may include additional information such as spectra and images to support refined association strategies. As data become increasingly structured, the agent’s tool layer will continue to expand, while the advanced decision-making capabilities of LLMs will enable flexible workflows beyond simple if-else logic and provide substantial potential for further extension.

## Toward agentic multi-messenger astronomy

This letter presents GW-Eyes, a first LLM-powered agent framework for GW counterpart association in multi-messenger astronomy. Through the design of Collector and Executor sub-agents and the integration of domain-specific tools, GW-Eyes enables EM catalog management, counterpart association analyses, and auxiliary support for human experts. Natural-language interaction significantly improves flexibility and provides interpretable reasoning traces that facilitate subsequent inspection and verification. In addition, GW-Eyes is highly extensible, allowing more specialized association strategies to be readily incorporated for different EM catalogs. We expect GW-Eyes to offer a promising approach to alleviate the growing human workload in the big-data era of multi-messenger astronomy.

## Data and code availability

The code of GW-Eyes is publicly available via GitHub at https://github.com/Yiming-astro/GW-Eyes. The repository provides instructions for downloading the data involved in this letter.

## Funding and acknowledgments

This work is supported by the 10.13039/501100004826Beijing Natural Science Foundation (1242018), the 10.13039/501100001809National Natural Science Foundation of China (12573042 and 123B2043), the National SKA Program of China (2020SKA0120300), the Max Planck Partner Group Program funded by the 10.13039/501100004189Max Planck Society, and the High-performance Computing Platform of 10.13039/100032554Peking University. This research has made use of data or software obtained from the Gravitational Wave Open Science Center (gwosc.org), a service of the LIGO Scientific Collaboration, the Virgo Collaboration, and KAGRA. The funders had no role in study design, data collection and analysis, decision to publish, or preparation of the manuscript.

## Author contributions

Y.D. designed and developed the project code, performed the main data analyses, and drafted the manuscript. Y.K. contributed to discussions and analyses related to multi-messenger astronomy. J.Z. contributed expertise in gravitational-wave counterpart associations. X.Z. contributed to agent development. Z.W. contributed expertise in gravitational-wave astronomy. L.S. contributed to project design and writing of the manuscript and provided supervision and coordination of the whole project. All authors contributed to the manuscript and approved the final version.

## Declaration of interests

The authors declare no conflicts of interest.

## Lead contact website

Lijing Shao: https://friendshao.github.io.
